# Selenium ameliorates *Staphylococcus aureus*-induced inflammation in bovine mammary epithelial cells by inhibiting activation of TLR2, NF-κB and MAPK signaling pathways

**DOI:** 10.1186/s12917-018-1508-y

**Published:** 2018-06-20

**Authors:** Heng Wang, Chongliang Bi, Yinjie Wang, Jun Sun, Xia Meng, Jianji Li

**Affiliations:** 1 College of Veterinary Medicine, Yangzhou University, Yangzhou, 225009 Jiangsu China; 2 Jiangsu Co-innovation Center for Prevention and Control of Important Animal Infectious Diseases and Zoonoses, Yangzhou, 225009 Jiangsu China; 3 College of Agriculture Forestry Science, Linyi University, Linyi, 276000 Shandong China

**Keywords:** Se, TLR2, NF-κB, MAPK, Mammary

## Abstract

**Background:**

*Staphylococcus aureus* (*S. aureus*) internalization into bovine mammary epithelial cells (bMECs) is considered an important pathogenic mechanism for the establishment of mastitis. Given the interesting link between selenium (Se) status and mastitis, our objective was to prove that Se was essential to suppress pro-inflammatory mediators, in part, by modulation of Toll-like receptor2 (TLR2), nuclear factor kappaB (NF-κB) and mitogen activated protein kinase (MAPK) signal transduction pathway in bMECs.

**Results:**

Results showed that Se (0~ 16 μM) did not affect the growth of bMECs. The mRNA expression of TLR2, Myeloid differentiation factor 88 (Myd88), Interleukin-1 receptor-associated kinase4 (Irak4), Interleukin-1 receptor-associated kinase1 (Irak1) and TNF receptor-associated factor6 (Traf6) in TLR2 signal pathway were increased or significantly increased by *S. aureus*. Se played an important role in regulating the genes expression of TLR2, Myd88, Traf6 but not in controlling the expression of Irak4 and Irak1. In addition, Se exerted strong inhibitory effects on the genes expression of tumor necrosis factor-alpha (TNF-α), interleukin-1β (IL-1β) and interleukin-6 (IL-6) induced by *S. aureus*. To further investigate the possible signaling mechanisms involved in the processes, we analyzed the role of MAPK and NF-κB signaling pathway in inflammation response in *S. aureus*-stimulated bMECs in vitro. Results showed that the phosphorylation of inhibitory kappaB alpha (*IκBα*), *p65*, *p38* and extracellular regulated protein kinase (*Erk*) were significantly increased in *S. aureus*-stimulated bMECs. It indicated that *S. aureus* activated NF-κB and MAPK signaling pathway. We also examined the effects of Se on the phosphorylation of *IκBα, p65, p38* and *Erk* in NF-κB and MAPK signaling pathway, which have well been proved to control the synthesis and release of pro-inflammatory mediators during inflammation. The findings are exciting, that pretreatment with Se (4, 8 μM) significantly suppressed the phosphorylation of *IκBα*, *p65*, *p38* and *Erk*.

**Conclusions:**

These results suggest that Se down-regulates inflammatory mediators TNF-α, IL-1β and IL-6 gene expressions via TLR2, NF-κB and MAPK signaling pathway in *S. aureus*-stimulated bMECs, which may be responsible for the anti-inflammatory effect of Se.

## Background

Bovine mastitis is a frequent and costly disease in the dairy industry [1]. *S. aureus* internalization into bMECs is considered an important pathogenic mechanism for the establishment of mastitis. In contrast to clinical mastitis, *S. aureus*-induced mastitis may manifest very diverse degrees of severity, from fulminating gangrenous mastitis with nervous systemic signs to mild local infection with only local signs [2, 3]. Therefore, it is of prime importance to determine how to detect and eradicate bacteria earlier and control the inflammation [4]. Antibiotics as an effective treatment of bovine mastitis existed many limits such as drug resistance and food safety concerns [5]. Thus, innate immune defense in local against pathogenic microorganisms has already attracted extensive attention.

The epithelium is an important line of defense against pathogenic microorganisms. Beyond the function of milk production, bMECs also equipped with a battery of receptors sensing the presence molecular components of pathogens, which has been verified that these cells are capable of initiating an in vitro innate immune response to pathogenic bacteria [6, 7]. Among these pattern recognition receptors (PPRs), 13 different TLRs have been well described in mammalian [8]. Some of these receptors span across the cell membrane and bind bacterial ligands with their extracellular domain. TLR2, for example, has been confirmed to be indispensable for *S. aureus* recognition [9]. Successful TLR2 signaling mostly involves the activation of the factor Myd88. Myd88 possesses two remarkable domains, the N-terminal death domain and C-terminal Toll/IL-1R domain (TIR) domain [10]. Ligand binding to the TLR family results in the recruitment of Myd88 to TIR domains of receptors. And the death domain of Myd88 is known to interact with Irak, a serine/threonine kinase harbors a death domain in N-terminal portion by homophilic interaction. Subsequently, another adaptor protein Traf6 is phosphorylated and recruited to Irak [11]. Further, the TLR2-*S. aureus* interaction leads to the rapid and coordinated activation of various intracellular signaling pathways such as NF-κB and MAPK [12]. NF-κB and MAPK signaling pathways, responsible for regulating the expression of cytokines, chemokines, are essential immune mediators during inflammation [13].

Se is an essential micronutrient, which has been investigated for various medical applications such as anti-bacterial, anti-oxidant, anti-inflammatory, and anti-cancer growth [14, 15]. Previous studies have shown the correlation between Se and LPS-induced mammary epithelial cells inflammatory response. Results demonstrated that Se exert beneficial effects to mammary epithelial cells, after the treatment of cells with the LPS. Result also showed that Se could inhibit *LPS*-induced inflammation response of mammary epithelial cells through the inhibition of NF-κB and MAPK activation [16]. However, the effect of Se in *S. aureus*-induced bovine mastitis is still unclear. The aim of this study was to investigate the protective effect of Se against *S. aureus*-induced inflammation injury in bMECs and to clarify the associated signaling pathways of Se supplement in *S. aureus*-infected bMECs.

## Methods

### bMECs isolation, cell culture, and treatment

bMECs were isolated from udders of lactating cows as previously described and cryopreserved in liquid nitrogen [17, 18]. Cows were from the University of Yangzhou herd and the protocol was approved by the Animal Care and Ethics Committee of the Yangzhou University (Approval ID: SYXK [Su] 2012–0029). Cells were cultured in Dulbecco’s modified Eagle’s medium/Ham’s F-12 nutrient mixture (DMEM/F12), supplemented with 10% fetal bovine serum (Gibco, Grand island), 5 μg/mL of insulin, 1 μg/mL of hydrocortisone, and 10 ng/mL of epidermal growth factor (Sigma, US).

Sodium selenite was diluted in DMEM/F12 medium. *S. aureus* (29,213, ATCC) was grown overnight at 37 °C in 10 mL Luria-Bertani. The number of *S. aureus* was determined by dilution method of plate counting. Bacteria was then diluted to achieve a multiplicity of infection (MOI) of 1:1 (bacteria:cell) in DMEM/F12. All cells were washed with serum-free medium and serum starved for 1 h before incubation with Se or *S. aureus*.

### Cell viability assay

To measure cell viability, 1 × 10^5^ cells of bMECs were seeded in 96-well multiplies and cultured in 100 μL DMEM/F12 medium at 37 °C and 5% CO_2_. When cells grew to 90% confluence, all cells were washed twice with phosphate buffer saline (PBS) and serum starved for 1 h before incubation with Se in different concentrations for 12 h, and then 10 μL of 3-(4,5-dimethylthiazol-2-yl)-2,5-diphenyltetrazolium bromide (Amresco, US) was added to each well and incubated for another 4 h. The formazan product was dissolved using dimethyl sulfoxide (Amresco, US). The optical density was measured at 570 nm using a microplate reader (Tecan, Austria) [19].

### RNA extraction and genes expression analysis

Total RNA was extracted from infected cells using Trizol reagent (Amresco, US) according to the manufacturer’s instructions. Integrity of RNA and reverse transcription were performed as previously described [20]. Reverse transcription-generated cDNA encoding β-actin, TLR2, Myd88, Irak4, Irak1, Traf6, IL-1β, TNF-α and IL-6 were amplified by RT-PCR using selective primers listed in Table 1. Quantitative PCR analysis was carried out as previously described [21]. The PCR reaction system contained 10 μL of SYBR Green PCR mix (Takara, Japan), 0.8 μL of each primer, 2 μL of cDNA template, and 6.4 μL of diethylpyrocarbonate water.

**Table 1:** Primers used in experiment

Gene	Sequence
β-actin	F:ACATCCGCAAGGACCTCTA R:CCATGCCAATCTCATCTCGTT
TNF-α	F:CTGCTGACGGGCTTTACC R:GACTGCAATGCGGCTGAT
IL-1β	F:GCTATGAGCCACTTCGTGAGGAC R:GATTGAGGGCGTCGTTCAGGAT
IL-6	F:TGATGACTTCTGCTTTCCCTACCC R:ATCTTTGCGTTCTTTACCCACTCG
TLR2	F:GATGACTACCGCTGTGAC R:GGTTTTGTGGCTCTTTTC
Myd88	F:AGCAGCATAACTCGGATAA R:CAGACACGCACAACTTCA
Irak4	F:TGGCAAAGACAGGACATCTG R:CACAACTCCCAAACCCTCCTT
Irak1	F:GAGTTCCAACGTCCTTCTGG R:CTCCCGGTCTTCACGTACTG
Traf6	F:CGGTGACTCTCTCCAGGTGT R:TGGACATTTGTGACCTGCAT

### Western blot analysis

Total protein was extracted from bMECs using a total protein extraction kit (BioChain, US) and the protein concentrations were determined by bicinchoninic acid protein assay kit (BioChain, US). All cells were respectively pretreated with Na_2_SeO_3_ (2, 4, and 8 μM) or left untreated for 12 h and infected with *S. aureus* (MOI = 1:1) for 0.5 h. Cell lysates were prepared as described previously [2]. Proteins were loaded into 10% SDS polyacrylamide gel for electrophoresis, and transferred using sodium phosphate buffer to polyvinylidene fluoride membrane metastasis (Merck Millipore, Germany). The membrane was then hybridized with specific antibodies. Antibodies included β-actin, phosphor-*IκBα* (*p-IκBα)*, phospho-*p65* (*p-p65*), phosphor-*p38* (*p-p38*), and phosphor-*Erk* (*p-Erk*) (Cell Signaling Technology, US).

### Statistical analysis

All data were presented as *mean ± standard error of mean* (sem). The groups were compared by using one-way ANOVA and Dunnett’s test. A *p*-value< 0.05 was considered statistically significant. Unless indicated otherwise, all data were obtained from at least 3 independent experiments.

## Results

### The cytotoxicity of se on bMECs

**Fig. 1:**
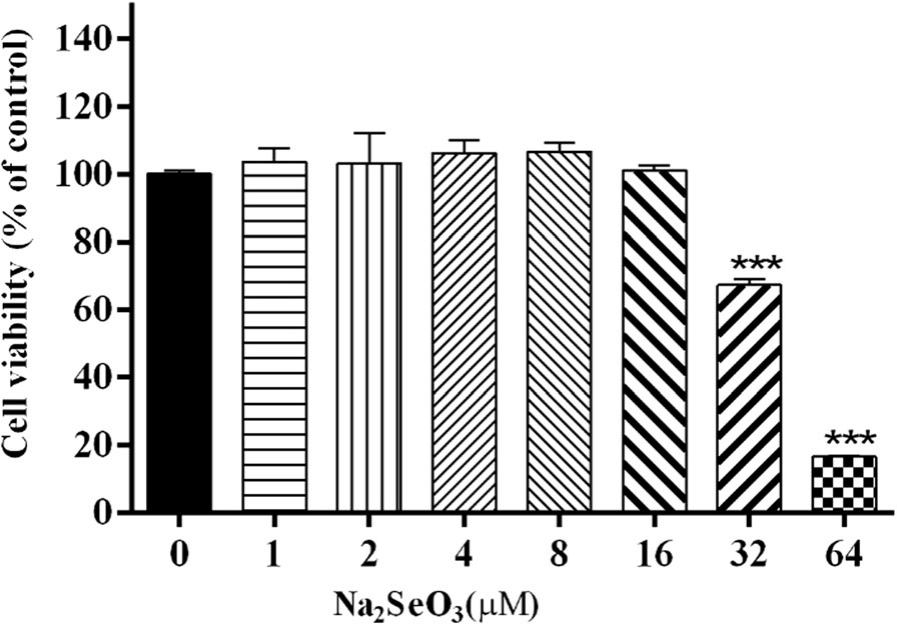
The cytotoxicity of Se on bMECs. Cell viability was measured by MTT following treatment with various concentrations (0, 1, 2, 4, 8, 16, 32 and 64 μM) of Se for 12 h. Cell proliferation was not inbitited by Se in concentration lower than 16 μM. The data are *mean ± sem* (*n* = 6). ^***^p < 0.001 vs. 0 μM

Results showed that there was no significant effect of 1~ 16 μmol/L selenium on cell activity. As selenium concentrations increased further, cell activity was significantly suppressed, compared with the control group (*p < 0.001*). Activity of bMECs fell by 33% with 32 μmol/L selenium, and by 84% with 64 μmol/L selenium, which shew cytotoxicity (Fig. 1).

### Se inhibits the activation of TLR2 pathway

**Fig. 2:**
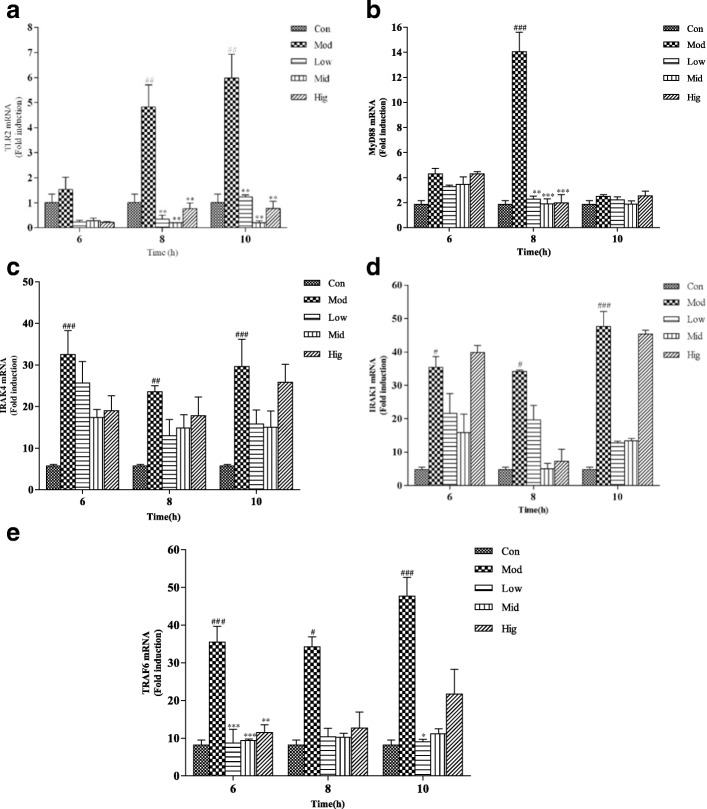
The effect of Se on TLR2 signaling pathway associate genes induced by *S. aureus* in bMECs. Cells were incubated with different concentrations of Se or serum-free medium for 12 h and subsequently treated with *S. aureus* (MOI = 1:1) for 0, 6, 8 and 10 h. Total RNA was prepared at the indicated time points after *S. aureus* treated. The TLR2 (**a**) , Myd88 (**b**) , Irak4 (**c**) , Irak1 (**d**) and Traf6 (**e**) mRNA expression levels were assayed using qRT-PCR. con = control cells without any treatment; mod = cells treated with *S. aureus* (MOI = 1:1) only; low = Se (2 μM) + *S. aureus* (MOI = 1:1); mid = Se (4 μM) + *S. aureus* (MOI = 1:1); high = Se (8 μM) + *S. aureus* (MOI = 1:1). The data are shown as *mean ± sem* (*n* = 3). ^*###*^*: p < 0.001* vs. Con; ^*^: p < 0.05 vs. Mod; ^**^: p < 0.01 vs. Mod; ^***^: p < 0.001 vs. Mod

As shown in Fig. 2a, the expression of TLR2 were up-regulated after *S. aureus* infection. This effect was significantly blocked by Se at 8 and 10 h in all group (*p < 0.01*). However at 6 h no statistically significant differences were shown between Se-pretreatment and control groups.

The genes expression of Myd88 induced by *S.aureus* were significantly up-regulated (*p < 0.001*) at 8 h and this effect was blocked by Se in all group (*p < 0.001*) (Fig. 2b). but at 6 h and 10 h there was no difference in statistics between all groups.

The gene expression of Irak4 induced by *S. aureus* were increased or significantly increased (*p < 0.01 or p < 0.001*) at all time point, but Se showed no blocking effect to the mRNA expression of Irak4 (Fig. 2c). Similar to Irak4, the mRNA expression of Irak1 were all up-regulated at any time points (*p < 0.05 or p < 0.001*) after *S. aureus* infection, and Se showed no blocking effect to the mRNA expression of Irak1 (Fig. 2d).

Compared to the control group, the Traf6 levels increased or significantly increased after *S. aureus* infection (*p < 0.05 or p < 0.001*) and this effect was blocked by Se in all group (*p < 0.001*) (Fig. 2e) at 6 h. So, the blocking effect of Se on TLR2 signal pathway in the inflammation response of bMECs induced by *S. aureus* was confirmed initially.

### Se weakens the inflammation injury of bMECs induced by *S. aureus*

**Fig. 3:**
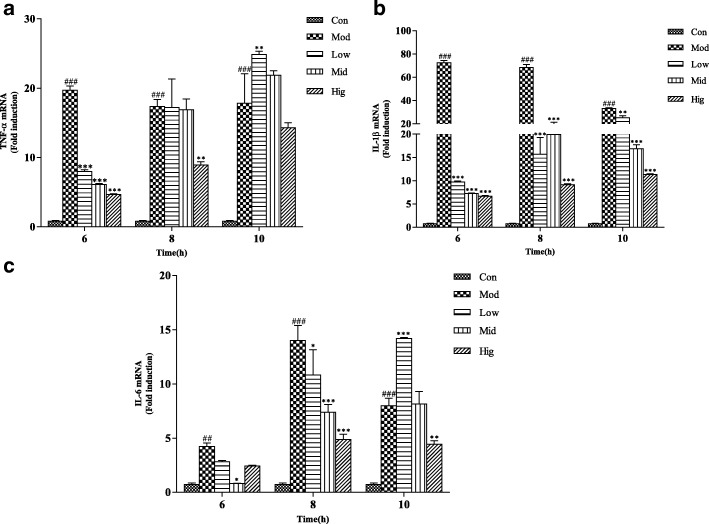
The effect of Se on gene expression of pro-inflammatory cytokines induced by *S. aureus* in bMECs. Cells were incubated with various concentrations of Se or serum-free medium for 12 h and subsequently challenged with *S. aureus* (MOI = 1:1) for 0, 6, 8, and 10 h. Total RNA was prepared at the indicated time points after *S. aureus* injection. The TNF-α, IL-1β and IL-6 mRNA expression were quantified using qRT-PCR. con = control cells without any treatment; mod = cells treated with *S. aureus* (MOI = 1:1) only; low = Se (2 μM) + *S. aureus* (MOI = 1:1); mid = Se (4 μM) + *S. aureus* (MOI = 1:1); high = Se (8 μM) + *S. aureus* (MOI = 1:1). The data are shown as *mean ± sem* (*n* = 3). ^*##*^*: p < 0.01* vs. Con; ^*###*^*: p < 0.001* vs. Con; ^*^: p < 0.05 vs. Mod; ^**^: p < 0.01 vs. Mod; ^***^: p < 0.001 vs. Mod

The protective effect of Se against *S. aureus*-induced inflammation injury in bMECs was analyzed by qRT-PCR. *S. aureus* could significantly up-regulate the gene expression of TNF-α, IL-1β, and IL-6 at the various time points. But the effect was markedly inhibited by Se (Fig. 3a, b and c). Thus, the anti-inflammatory effect of Se was confirmed in the inflammation response of bMECs induced by *S. aureus*.

### Se inhibits the activation of NF-κB pathway

**Fig. 4:**
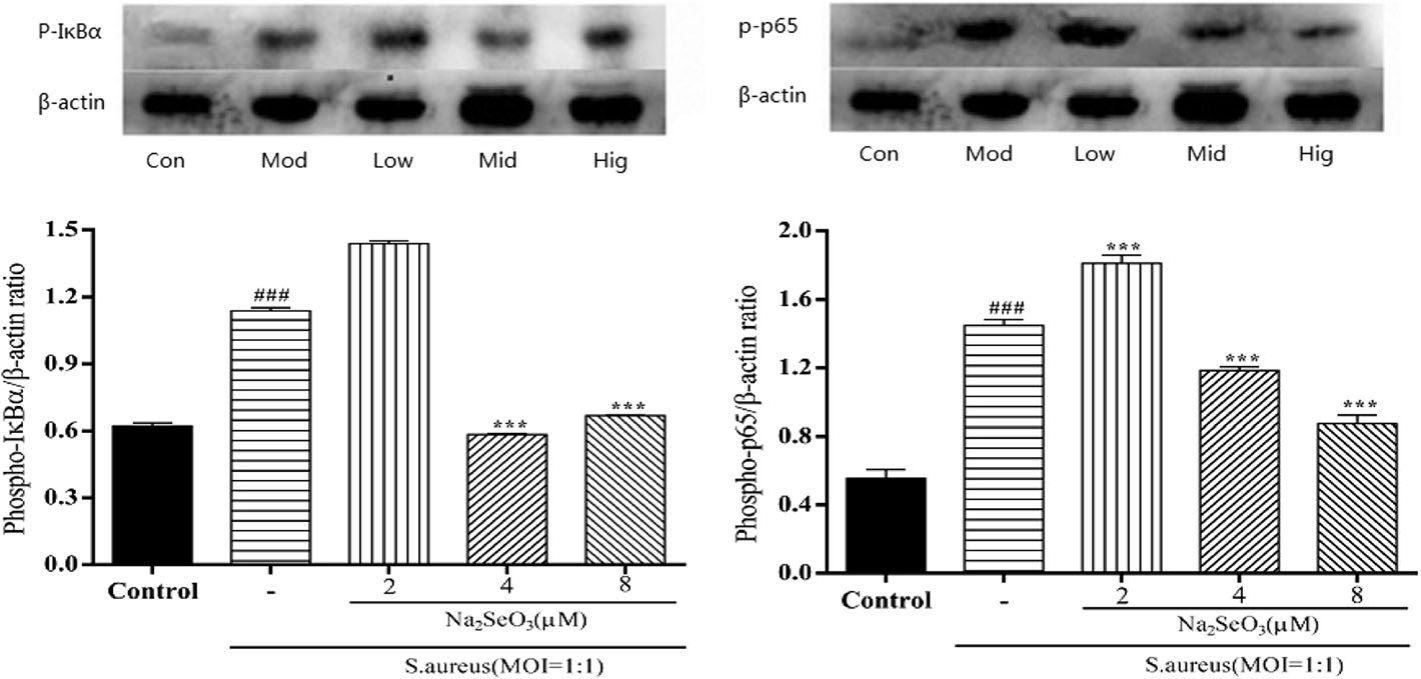
Effect of Se on *S. aureus*-induced IκBα and p65 phosphorylation in bMECs. Cells were pretreated with various concentrations (0, 2, 4 and 8 μM) of Se or serum-free medium for 12 h before stimulated with *S. aureus* (MOI = 1:1) for 0.5 h and then washing twice with PBS. Total proteins were prepared at the indicated time points and subjected to Western blotting. Con = control cells without any treatment; mod = cells treated with *S. aureus* (MOI = 1:1) only; low = Se (2 μM) + *S. aureus* (MOI = 1:1); mid = Se (4 μM) + *S. aureus* (MOI = 1:1); high = Se (8 μM) + *S. aureus* (MOI = 1:1). The data are shown as *mean ± sem* (n = 3). ^*###*^*: p < 0.001* vs. Con; ^*^: p < 0.05 vs. Mod; ^**^: p < 0.01 vs. Mod; ^***^: p < 0.001 vs. Mod. One out of 3 independent experiments is shown

Protein expression of *p-IκBα* and *p-p65* were up-regulated significantly (*p < 0.001*) by innoculation with *S. aureus* for 0.5 h (Fig. 4), which indicated that NF-κB signaling pathway was activated. However, the phosphorylation of *IκBα* and *p65* were suppressed by the addition of 4 μM and 8 μM Se. So, the higher Se concentration could suppress the inflammatory response by NF-κB pathway to some extent.

### Se inhibits the activation of MAPK pathway

**Fig. 5:**
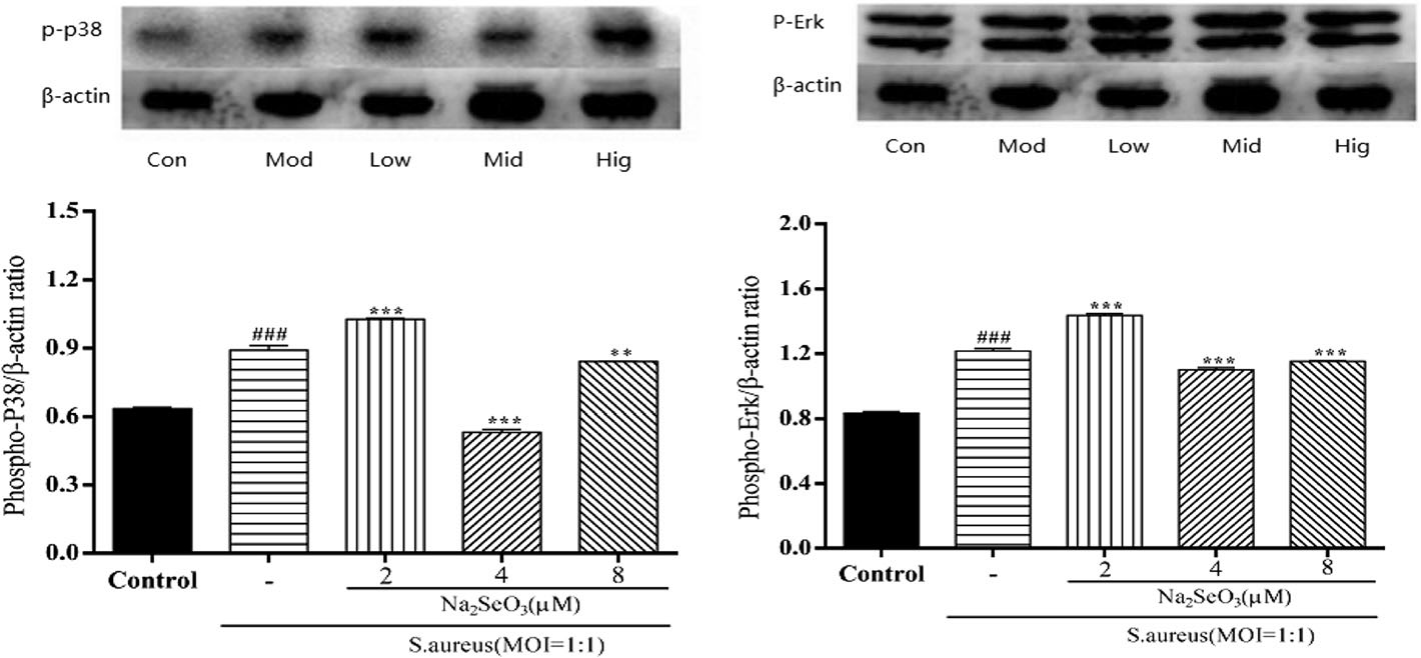
Effect of Se on *S. aureus*-induced p38 and Erk phosphorylation in bMECs. Cells were pretreated with various concentrations (0, 2, 4 and 8 μM) of Se or serum-free medium for12 h before stimulated with *S. aureus* (MOI = 1:1) for 0.5 h and then washing twice with PBS. Total proteins were prepared at the indicated time points and subjected to Western blotting. Con = control cells without any treatment; mod = cells treated with *S. aureus* (MOI = 1:1) only; low = Se (2 μM) + *S. aureus* (MOI = 1:1); mid = Se (4 μM) + *S. aureus* (MOI = 1:1); high = Se (8 μM) + *S. aureus* (MOI = 1:1). The data are shown as *mean ± sem* (n = 3). ^*###*^*: p < 0.001* vs. Con; ^*^: p < 0.05 vs. Mod; ^**^: p < 0.01 vs. Mod; ^***^: p < 0.001 vs. Mod. One out of 3 independent experiments is shown

The phosphorylation of *p38* and *Erk* increased significantly with *S. aureus* or associated with 2 μM Se innoculation for 0.5 h, while phosphorylation of *p38* and *Erk* were markedly suppressed by Se at 4 and 8 μM (Fig. 5), which suggesting that higher concentration of Se could inhibit the activation of MAPK signaling pathway by suppression of phosphorylation of *p38* and *Erk*.

## Discussion

Se is an essential micronutrient that play an important role in regulating the immune function [22]. Many studies have indicated that Se is involved in several biological activities, including the regulation of inflammation and immunological reaction [23]. The acute and sub-actue toxicity of Se were studies in bMECs, and these results showed that when the concentrations of Se is lower than 16 μM, the cell viability was not affected, which is also within the normal range of selenium in the dairy cow.

Our previous study supports the hypothesis that TLR2 plays an important role at the early inflammation induced by *S. aureus* [24]. In the meanwhile, Irak was activated via an adaptor protein Myd88 in TLR2 signalling pathway [25, 26]. In this study, proteins expression of TLR2 signaling pathway were examined by qRT-PCR. Accompanied by the activation of Irak, another adaptor protein Traf 6 is phosphorylated and recruited to Irak in model group. Then, NF-κB and MAPK signaling pathway were triggered, which were characterized by the releasing of a large number proinflammatory cytokines [27]. Appropriate inflammation response is beneficial for immune cells to fight against infection, but excessive inflammation response will injure the tissues and cells. So the releasing of proinflammatory cytokines must be tightly regulated during the inflammatory response [28]. Our study found that a strong activity of TLR2 signaling pathway could be triggered by *S. aureus* at about 10 h. Moreover, the gene expression of TNF-α, IL-1β, and IL-6 were significantly increased in bMECs induced by *S. aureus* at any time point, which indicated a strong inflammatory response. Se obviously suppressed the mRNA expression of TLR2, Myd88, and Traf6 induced by *S. aureus* at 6, 8, 10 h. The results support the hypothesis that Se could protect bMECs from *S. aureus* injury by inhibiting the expression of TNF-α, IL-6 and IL-1β. Furthermore the effect of Se on TLR2 signaling pathway is mainly regulated by the gene expression of TLR2, Myd88 and Traf6.

NF-κB and MAPK signaling pathways are two classical pathways associated with inflammation, which are important in numerous processes such as immune processes, cell survival and inflammation [29, 30]. As an accelerant of NF-κB and MAPK, *S. aureus* could strongly enhance the phosphorylation of *IκBα, p65, p38*, and *Erk*, leading to a conformational change of NF-κB and MAPK that evokes NF-κB and MAPK signaling activity. The different members of NF-κB and MAPK have different downstream targets, and thus play distinct roles in cellular responses. Our results showed that the phosphorylation of *IκBα, p65, p38,* and *Erk* were markedly increased after stimulation with *S. aureus* for 0.5 h., which demonstrated a significant activation of NF-κB and MAPK and subsequently lead to a high expression of pro-inflammation cytokines. Se suppressed the phosphorylation of *IκBα, p65, p38, Erk*, which indicating an inhibitory effect of NF-κB and MAPK activity. Therefore, the releasing of pro-inflammatory cytokines were inhibited. Ultimately, the protective effect of Se was validated through blocking TLR2, NF-κB and MAPK signaling pathways against bMECs inflammatory injury induced by *S. aureus*.

## Conclusions

This study proved the protective effect of Se on *S. aureus*-induced inflammation in bMECs. This effect was at least partly achieved by the blocking of TLR2 signaling pathway. On the other hand, Se could decrease the gene expression of pro-inflammation cytokines through mediating the phosphorylation of IκBα and p65 in NF-κB pathway and inhibiting the phosphorylation of p38 and Erk in the MAPK pathway. Se reveal potential benefit for the adjuvant therapy of mastitis induced by *S. aureus*. But other anti-inflammatory associated pathways and targets of Se need to be studied in the future.

## Abbreviations

bMECS: Bovine mammary epithelial cells
MAPK: Mitogen-activated protein kinase
NF-κB: Nuclear factor kappa-light-chain-enhancer of activated B cells.
Se: Selenium.
TLR2: Toll-like receptor 2.
